# Myosin di-phosphorylation and peripheral actin bundle formation as initial events during endothelial barrier disruption

**DOI:** 10.1038/srep20989

**Published:** 2016-02-11

**Authors:** Mayumi Hirano, Katsuya Hirano

**Affiliations:** 1 Department of Molecular Cardiology, Research Institute of Angiocardiology, Graduate School of Medical Sciences, Kyushu University.; 2 Department of Cardiovascular Physiology, Faculty of Medicine, Kagawa University.

## Abstract

The phosphorylation of the 20-kD myosin light chain (MLC) and actin filament formation play a key role in endothelial barrier disruption. MLC is either mono- or di-phosphorylated (pMLC and ppMLC) at T18 or S19. The present study investigated whether there are any distinct roles of pMLC and ppMLC in barrier disruption induced by thrombin. Thrombin induced a modest bi-phasic increase in pMLC and a robust mono-phasic increase in ppMLC. pMLC localized in the perinuclear cytoplasm during the initial phase, while ppMLC localized in the cell periphery, where actin bundles were formed. Later, the actin bundles were rearranged into stress fibers, where pMLC co-localized. Rho-kinase inhibitors inhibited thrombin-induced barrier disruption and peripheral localization of ppMLC and actin bundles. The double, but not single, mutation of phosphorylation sites abolished the formation of peripheral actin bundles and the barrier disruption, indicating that mono-phosphorylation of MLC at either T18 or S19 is functionally sufficient for barrier disruption. Namely, the peripheral localization, but not the degree of phosphorylation, is suggested to be essential for the functional effect of ppMLC. These results suggest that MLC phosphorylation and actin bundle formation in cell periphery are initial events during barrier disruption.

Vascular endothelial cells form a monolayer that lines the luminal surface of the vasculature, and these play a critical role in regulating the transport of materials between the vascular lumen and extravascular spaces. The regulated endothelial barrier function is attributable to two mechanisms; paracellular and transcellular pathways^1^,^2^. Under physiological conditions, particles larger than approximately 3 nm in radius, such as serum albumin, are transported through the transcellular pathway, while the smaller molecules, such as water, ions or glucose, permeates through paracellular pathway according to Fick’s law^1^,^2^. The integrity of the endothelial barrier function plays an important role in maintaining vascular homeostasis. The dysregulation of the endothelial barrier function is not only a hallmark of acute inflammation but also an important predisposing factor for the pathogenesis of various vascular diseases, including atherosclerosis, diabetic vasculopathy, acute pulmonary injury or pulmonary hypertension^1^,^2^,^3^,^4^. The disruption of the paracellular pathway plays a central role in endothelial barrier dysfunction.

The VE-cadherin-mediated adherens junction, together with tight junction (especially in the case of the cerebral artery), is an essential component of inter-endothelial junctions that play a critical role in regulating the paracellular barrier function^1^,^2^,^3^,^4^. The disruption of the inter-endothelial junctions and the resultant gap formation are clear manifestations of endothelial barrier dysfunction. In addition to impairment of the function of inter-endothelial junctions, the phosphorylation of 20-kD myosin light chain (MLC) and the resultant actin filament formation also play critical roles during barrier dysfunction by providing the force to disrupt the inter-endothelial junctions^1^,^2^,^3^,^4^. The molecular mechanisms underlying physiological barrier formation and pathological barrier disruption have been intensively studied using cultured endothelial cells. At confluence, the quiescent cells are characterized by a continuous VE-cadherin lining associated with circumferential actin bundles, and a low level of MLC phosphorylation with sparse actin stress fibers. Increased activity of a small G protein, Rac1, and low activity of RhoA are also associated with highly confluent endothelial cells^1^,^2^,^3^,^4^,^5^. In contrast, various factors such as thrombin, lipopolysaccharide and vascular endothelial growth factor cause barrier disruption by increasing RhoA activity, MLC phosphorylation and actin stress fiber formation^1^,^2^,^3^,^4^,^5^. The disassembly of circumferential actin bundles and development of actin stress fibers are characteristic of endothelial cells with impaired barrier function^2^,^5^. However, it remains unclear how this rearrangement of actin filaments from the circumferential bundle to the stress fibers takes place during barrier disruption.

MLC is phosphorylated at multiple sites^6^,^7^,^8^,^9^. Among them, T18 and S19 are the phosphorylation sites associated with an increase in myosin ATPase activity, the formation of actin filaments such as stress fibers, the stabilization of myosin filaments and cellular contraction, migration and cytokinesis^6^. Ca^2+^-calmodulin-dependent MLC kinase (MLCK) is the first kinase that was identified to phosphorylate T18 and S19^6^,^10^. MLCK phosphorylates MLC with preference for S19 over T18; therefore, the phosphorylation of S19 and T18 takes place in a sequential manner^6^,^11^,^12^. Later, other kinases including Rho-kinase, Zipper-interacting kinase and integrin-linked kinase were also identified to phosphorylate MLC with no preference between T18 and S19^13^,^14^,^15^.

The functional differences between mono-phosphorylated and di-phosphorylated MLC (pMLC and ppMLC) are known to be associated with the regulation of myosin ATPase activity, actin filament formation, stabilization of myosin filaments, cytokinesis, cellular stiffness and cellular migration^11^,^12^,^16^,^17^,^18^,^19^,^20^,^21^,^22^,^23^. However, whether pMLC and ppMLC play any differential role in endothelial barrier disruption still remains to be investigated.

Thrombin is a serine proteinase that plays a key role in the blood coagulation. Thrombin is also known as a potent inducer of endothelial barrier disruption^1^,^2^,^3^. The cellular effects of thrombin are mediated by a unique family of G protein-coupled receptor, referred to as proteinase-activated receptor (PAR)^24^,^25^. Among four subtypes of PAR, PAR_1_, PAR_3_ and PAR_4_ serve as receptors for thrombin. PAR_1_ and PAR_3_ have higher affinity for thrombin than PAR_4_, while PAR_3_ lacks signaling activity^24^,^25^. Therefore, PAR_1_ serves as a high-affinity signaling receptor for thrombin, and plays major role in the vascular effects of thrombin^24^,^25^.

The present study aimed to elucidate whether there are any functional differences between pMLC and ppMLC, with regard to the formation of actin filaments during thrombin-induced barrier disruption by using cultured porcine aortic endothelial cells. The levels of pMLC and ppMLC were separately quantified with Phos-tag SDS-PAGE followed by immunoblot detection, as described previously^26^,^27^. The present study demonstrates, for the first time, that pMLC and ppMLC play distinct roles during endothelial barrier disruption. Furthermore, ppMLC and actin filament formation at the cell periphery play critical roles as initial events during thrombin-induced barrier disruption. The stress fiber formation associated with pMLC was observed in the late phase of barrier disruption. The observations described in the present study may thus provide a new viewpoint on the mechanism of endothelial barrier disruption.

## Results

### Proteinase-activated receptor 1 (PAR_1_)-mediated barrier disruption by thrombin in porcine aortic endothelial cells (PAECs)

**Figure 1:**
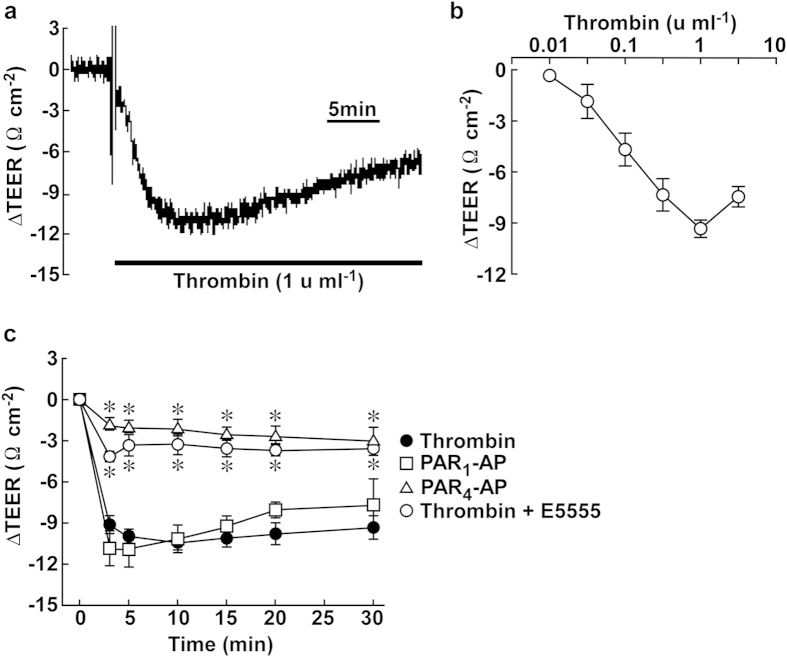
Thrombin-induced barrier disruption in PAECs. (**a**) A representative trace of the changes in the TEER induced by 1 u ml^−1^ thrombin. (**b**) The concentration-dependent effects of thrombin on the TEER at 3 min after stimulation (n = 3–7). (**c**) A summary of the temporal changes in the TEER induced by 1 u ml^−1^ thrombin without (Thrombin; n = 12) and with 1 μM E5555 (Thrombin + E5555; n = 4), 30 μM PAR_1_-AP (n = 4) and 30 μM PAR_4_-AP (n = 6). The data are the means ± SEM. *P < 0.05 vs. thrombin, according to an ANOVA followed by Dunnett’s post-hoc test.

Thrombin stimulation induced a sustained decrease in the trans-endothelial electrical resistance (TEER), which showed a maximum decrease at 3–5 min after thrombin stimulation (Fig. 1a). The barrier disrupting effect of thrombin was observed at concentrations higher than 0.01 u ml^−1^, and the maximum effect was obtained with 1 u ml^−1^ thrombin (Fig. 1b). PAR_1_ and PAR_4_ are the signaling receptors for thrombin^24^,^25^. PAR_1_-activating peptide (PAR_1_-AP; 30 μM) induced a decrease in the TEER to a level comparable to that seen following treatment with 1 u ml^−1^ thrombin, while PAR_4_-AP had no significant effect (Fig. 1c). The thrombin-induced decrease in the TEER was abolished by treatment with 1 μM of a PAR_1_ antagonist, E5555 (Fig. 1c). These observations indicated that thrombin induces barrier disruption mainly through PAR_1_ in PAECs.

### Thrombin-induced MLC phosphorylation in PAECs

**Figure 2:**
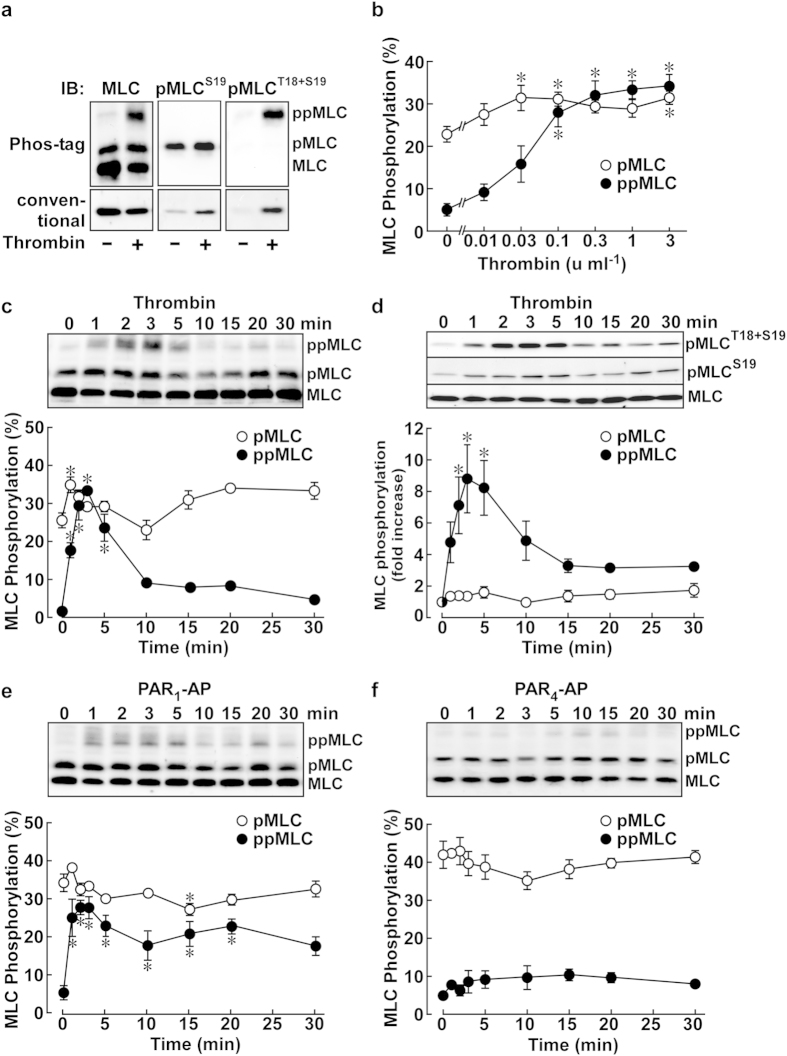
Thrombin-induced phosphorylation of MLC in PAECs. (**a**) Representative immunoblots (IB) after Phos-tag and conventional SDS-PAGE. The samples obtained before (−) and 3 min after (+) stimulation with 1 u ml^−1^ thrombin were applied, in triplicate, to SDS-PAGE. Each set of samples was probed with the indicated primary antibodies. MLC, non-phosphorylated MLC; pMLC, mono-phosphorylated MLC, ppMLC, di-phosphorylated MLC. (**b**) The concentration-dependent effects of thrombin on pMLC and ppMLC 3 min after stimulation (n = 4–7). (**c**–**f**) Representative immunoblots and a summary of the temporal changes in the levels of pMLC and ppMLC induced by 1 u ml^−1^ thrombin (**c**; n = 4, **d**; n = 4–7), 30 μM PAR_1_-AP (**e**; n = 4–5) and 30 μM PAR_4_-AP (**f**; n = 4), as indicated. The samples were analyzed with Phos-tag (**c**,**e**,**f**) or conventional (**d**) SDS-PAGE. In (**d**) the samples were subjected to SDS-PAGE in triplicate, followed by detection with the indicated primary antibody. The data are the means ± SEM. *P < 0.05 vs. the value obtained without thrombin stimulation (**b**) and at time 0 (**c**–**f**), according to an ANOVA followed by Dunnett’s post-hoc test.

The MLC phosphorylation was analyzed with Phos-tag SDS-PAGE followed by immunoblotting with an anti-pan MLC antibody. The anti-pan MLC antibody detected three bands in PAECs both before and after thrombin stimulation (Fig. 2a, Phos-tag, IB: MLC). The lower, middle and upper bands ran as the size of 20 kD, 23 kD and 29 kD, respectively, according to the mobility of the molecular weight markers (New England BioLabs, Ipswich, MA, USA). The density of the uppermost band apparently increased, while the density of the lowest band decreased after thrombin stimulation. The middle band was also recognized by the anti-pMLC^S19^ antibody, while the upper band was recognized by the anti-pMLC^T18+S19^ antibody (Fig. 2a, Phos-tag). Accordingly, the lower, middle and upper bands represent non-, mono- and di-phosphorylated MLC, respectively. Furthermore, these observations corroborated the mono-specificity of the anti-pMLC^S19^ and anti-ppMLC^T18+S19^ antibodies, which was essential for the immunofluorescence staining in the subsequent investigations.

In conventional SDS-PAGE, MLC ran as a single band (Fig. 2a, conventional). The amounts of total MLC were similar before and after thrombin stimulation, while the levels of pMLC and ppMLC apparently increased after thrombin stimulation (Fig. 2a, conventional, IB: pMLC^S19^, pMLC^T18+S19^). When evaluated 3 min after thrombin stimulation, there was a concentration-dependent increase in ppMLC, and the maximum effect was obtained around 0.3 u ml^−1^ thrombin (Fig. 2b). ppMLC was scarcely detected before thrombin stimulation (Fig. 2c). However, upon stimulation with 1 u ml^−1^ thrombin, the level of ppMLC substantially increased to a peak around 3 min after stimulation, and gradually decreased to the prestimulation level thereafter (Fig. 2c). The PAECs contained 25% pMLC before stimulation. The level of pMLC slightly increased after thrombin stimulation, and exhibited a bi-phasic response, with a dip at 10 min (Fig. 2c). However, the changes in pMLC were modest. Similar changes in pMLC and ppMLC were observed with conventional SDS-PAGE followed by three separate detections with the anti-pan MLC, pMLC^S19^, and pMLC^T18+S19^ antibodies (Fig. 2d). PAR_1_-AP also induced changes in pMLC and ppMLC similar to those seen with thrombin, while PAR_4_-AP had no significant effect (Fig. 2e,f).

### Effects of Rho-kinase inhibitors and the removal of extracellular Ca^2+^ on the thrombin-induced MLC phosphorylation and barrier disruption in PAECs

**Figure 3:**
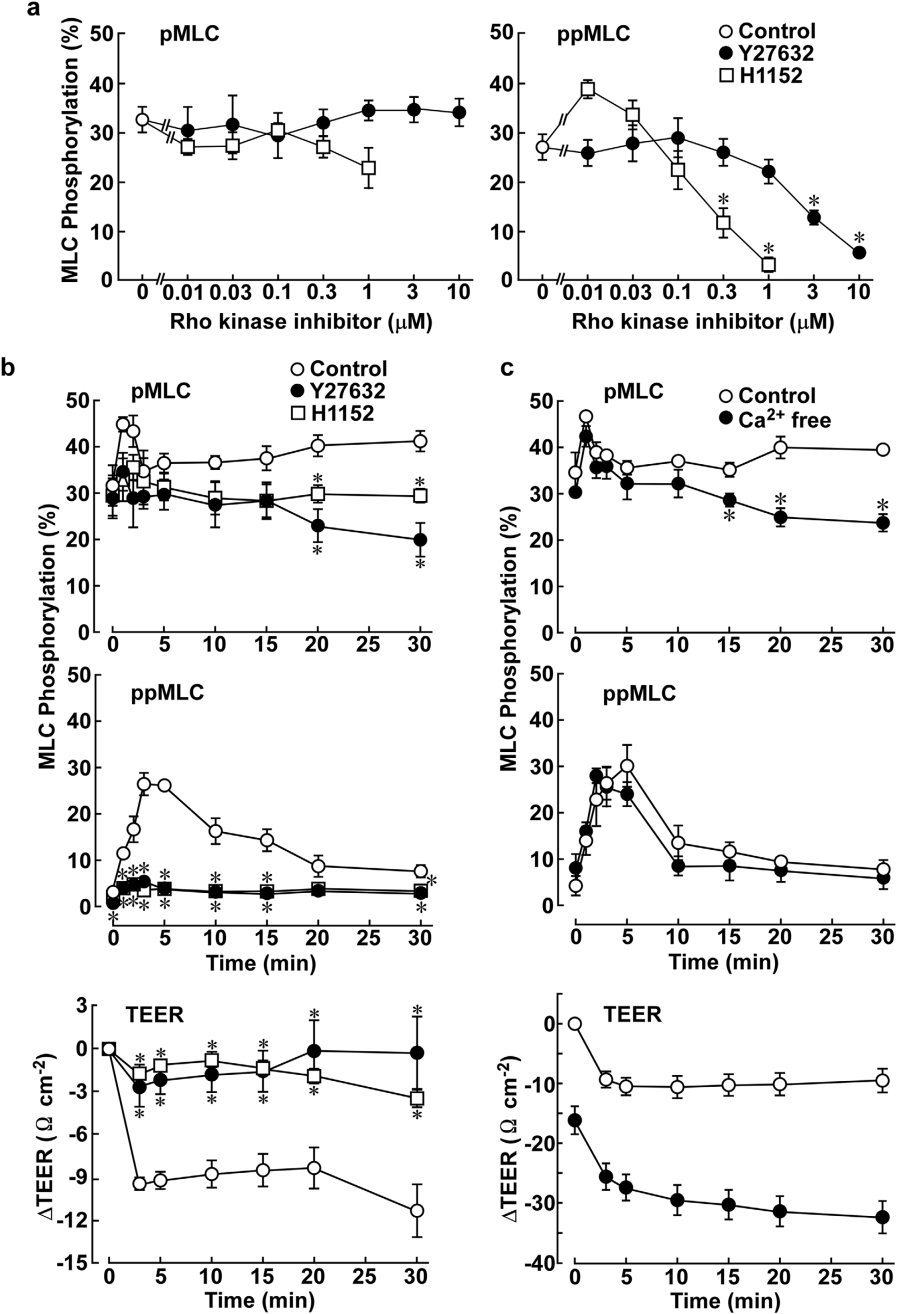
The effects of Rho-kinase inhibitors and the removal of extracellular Ca^2+^ on the thrombin-induced MLC phosphorylation and decrease in the TEER in PAECs. (**a**) The concentration-dependent effects of Y27632 (0.01–10 μM; n = 4–6) and H1152 (0.01–1 μM; n = 4) on pMLC and ppMLC 3 min after stimulation with 1 u ml^−1^ thrombin (Control: n = 10). The control values were those obtained 3 min after thrombin stimulation in the absence of inhibitors. (**b**) The temporal changes in pMLC and ppMLC (n = 4) and the TEER (n = 6 for control; n = 5 for Y27632; n = 4 for H1152) after stimulation with 1 u ml^−1^ thrombin in the absence and presence of 10 μM Y27632 or 1 μM H1152. (**c**) The temporal changes in pMLC and ppMLC (n = 4) and the TEER (n = 5) after stimulation with 1 u ml^−1^ thrombin in the presence and absence of extracellular Ca^2+^. PAECs were exposed to Y27632, H1152 or Ca^2+^-free HBS 30 min prior to and during thrombin stimulation. The data are the means ± SEM. *P < 0.05 vs. control, according to an ANOVA followed by Dunnett’s post-hoc test.

Rho-kinase and Ca^2+^ signaling are the two main signaling pathways leading to the phosphorylation of MLC^2^,^14^,^15^. Their roles in the thrombin-induced MLC phosphorylation were examined (Fig. 3). Rho-kinase inhibitors, Y27632 and H1152, were applied 30 min prior to and during stimulation with 1 u ml^−1^ thrombin. The resting level of ppMLC slightly decreased during the pretreatment with Y27632 (Fig. 3b, time 0); otherwise, no significant effects were observed for the resting levels of pMLC or ppMLC. However, both Y27632 and H1152 concentration-dependently inhibited the increase in ppMLC observed 3 min after thrombin stimulation (Fig. 3a). Almost complete inhibition of ppMLC was obtained with 10 μM Y27632 and 1 μM H1152. However, these inhibitors had no significant effect on the levels of pMLC. During the time course after thrombin stimulation, Y27632 (10 μM) and H1152 (1 μM) almost completely abolished the increase in ppMLC (Fig. 3b, ppMLC). The increase in pMLC seen at 3 min appeared to be inhibited by H1152 and Y27632; however, their inhibitory effects were not statistically significant. In contrast, Y27632 and H1152 consistently and significantly decreased the level of pMLC seen in the late phase, i.e., 15–30 min after thrombin stimulation (Fig. 3b, pMLC). The pretreatment with Y27632 and H1152 had no significant effect on the resting TEER; however, they almost completely abolished the thrombin-induced decrease in the TEER (Fig. 3b, TEER).

The removal of extracellular Ca^2+^ had no significant effect on the resting levels of both pMLC and ppMLC (Fig. 3c, time 0). The early phase (3 min) of pMLC was resistant to the Ca^2+^ removal, while the late phase (>10 min) significantly decreased in the absence of extracellular Ca^2+^ (Fig. 3c, pMLC). However, the thrombin-induced increase in ppMLC was resistant to Ca^2+^ removal (Fig. 3c, ppMLC). When the extracellular Ca^2+^ was removed, the resting TEER gradually decreased during the 30-min incubation (Fig. 3c, TEER). The subsequent thrombin stimulation induced a decrease in the TEER similar to that observed in the presence of extracellular Ca^2+^ (Fig. 3c, TEER).

As a result, the level of ppMLC, but not pMLC, was correlated with the degree of barrier disruption induced by thrombin. pMLC and ppMLC exhibited different sensitivities toward Rho-kinase inhibition and the removal of extracellular Ca^2+^.

### Effects of a myosin ATPase inhibitor, blebbistatin, on thrombin-induced and barrier disruption in PAECs

The effect of a myosin ATPase inhibitor, blebbistatin, on the thrombin-induced decrease in the TEER was examined to address the involvement of myosin-based contraction in the barrier disruption^28^. Pretreatment with 100 μM blebbistatin 30 min prior to and during thrombin stimulation substantially inhibited the thrombin-induced decrease in the TEER (see supplementary Fig. S1). However, blebbistatin had no effect on the levels of pMLC and ppMLC either before or 3 min after thrombin stimulation (see supplementary Fig. S1).

### Distinct subcellular localization of pMLC and ppMLC after thrombin stimulation in confluent PAECs

**Figure 4:**
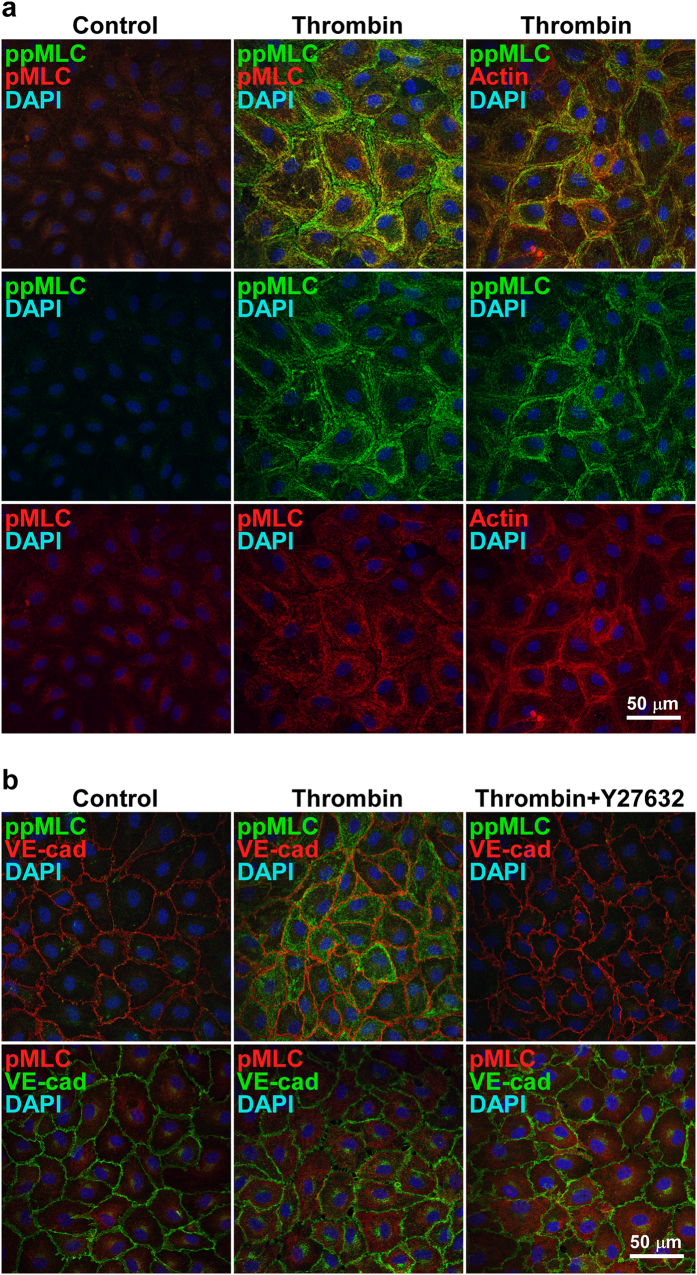
The localization of pMLC, ppMLC and actin in PAECs 3 min after thrombin stimulation with and without a Rho-kinase inhibitor. (**a**) Representative confocal microphotographs showing the localization of pMLC, ppMLC and actin before (Control) and 3 min after stimulation with 1 u ml^−1^ thrombin (Thrombin). Left (Control) and middle (Thrombin) columns; n = 7, right column (Thrombin); n = 2. The top panels show merged images of corresponding middle and bottom panels. (**b**) Representative confocal microphotographs showing the localization of pMLC and ppMLC in relation to VE-cadherin (VE-cad) before (Control) and 3 min after stimulation with 1 u ml^−1^ thrombin in the absence (Thrombin) and presence (Thrombin + Y27632) of 10 μM Y27632. Upper row; n = 3, Lower row; n = 2.

The mono- and di-phosphorylation of MLC were originally considered to be sequential events in smooth muscle cells^6^,^12^. However, the observations of the present study suggest that there are distinct regulatory pathway and functional roles of pMLC and ppMLC during thrombin-induced barrier disruption in PAECs. To confirm this finding, any distinct subcellular localization of pMLC and ppMLC and its correlation with actin filament formation was examined (Fig. 4). Before thrombin stimulation, pMLC was observed homogeneously in the perinuclear cytoplasmic region, while ppMLC was scarcely detected (Fig. 4a, left column). Three minutes after thrombin stimulation, intense staining of ppMLC was characteristically detected in the peripheral region of the cells (Fig. 4a, middle column). The intensity of pMLC appeared to increase; however, its localization remained unchanged (Fig. 4a, middle column). As a result, the localization of pMLC and ppMLC after thrombin stimulation was distinct, represented by central pMLC staining, surrounded by peripheral ppMLC staining (Fig. 4a, the middle column, top panel). The distinct localization of pMLC and ppMLC was also supported by double staining with VE-cadherin (Fig. 4b, left and middle columns). ppMLC was closely associated with VE-cadherin staining, while some separation was observed between pMLC and VE-cadherin. The double staining of ppMLC and actin revealed their colocalization in a peripheral bundle pattern (Fig. 4a, right column). Notably, actin did not show up in a stress fiber pattern 3 min after thrombin stimulation. The pretreatment with 10 μM Y27632 abolished the peripheral localization of ppMLC 3 min after thrombin stimulation, while the central cytoplasmic localization of pMLC was not affected by Y27632 treatment (Fig. 4b, right column).

### Thrombin induces stress fiber formation in PAECs with loose cell-cell adhesion

**Figure 5:**
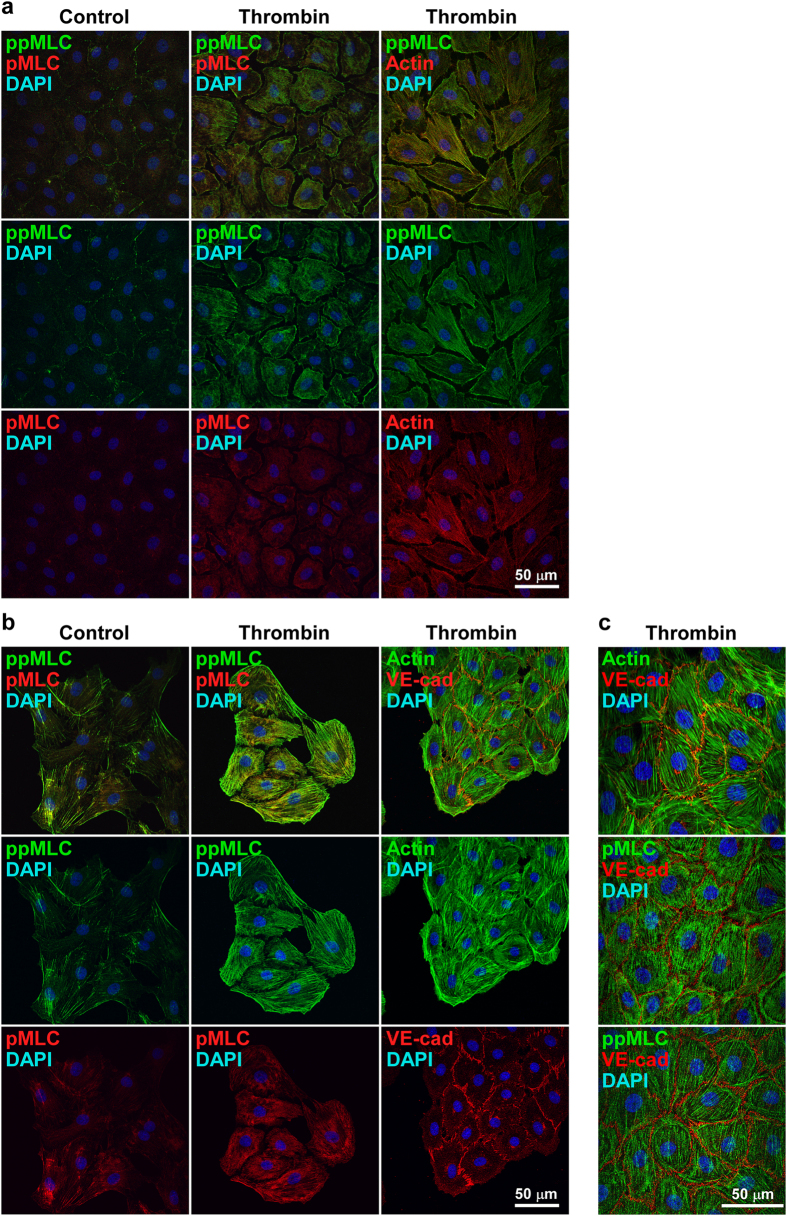
The stress fiber pattern of the localization of pMLC, ppMLC and actin 3 min after thrombin stimulation in PAECs, in the absence of extracellular Ca^2+^ at confluence or on an early culture day. Representative confocal microphotographs showing pMLC, ppMLC, actin or VE-cadherin (VE-cad), as indicated, before (Control) and 3 min after stimulation with 1 u ml^−1^ thrombin (Thrombin) in the absence of extracellular Ca^2+^ at confluence ((**a**), n = 2 for left and middle columns, n = 1 for right column) and on culture day 3 ((**b**), low density area, n = 2 for the left and middle columns, n = 3 for the right column; (**c**), high density area, n = 3 for all staining experiments). In (**a**,**b**) the top panels show merged images of corresponding middle and bottom panels.

The observations in Fig. 4 were apparently inconsistent with those in previous reports, which showed stress fiber formation after thrombin stimulation^1^,^2^,^3^,^4^. The difference in the state of cell-cell adhesion could be one possible explanation for this discrepancy. Therefore, the localization of pMLC, ppMLC and actin filaments was examined in PAECs with loose cell-cell adhesion (Fig. 5). Such cells were prepared by removing the extracellular Ca^2+^ in the confluent culture or on an early culture day (day 3) after plating.

pMLC and ppMLC were scarcely detected after a 30-min incubation in the absence of extracellular Ca^2+^ and before thrombin stimulation (Fig. 5a, left column). Upon thrombin stimulation, ppMLC showed a stress fiber pattern in 3 min, which was co-localized with pMLC (Fig. 5a, middle column) as well as actin filaments (Fig. 5a, right column). On culture day 3, pMLC and ppMLC exhibited sparsely filamentous co-localization in a stress fiber pattern before thrombin stimulation in the area of a low cell density (Fig. 5b, left column), where the lining of VE-cadherin was less developed (Fig. 5b, right column). Upon thrombin stimulation, intense co-staining of pMLC and ppMLC in a stress fiber pattern was observed 3 min after stimulation (Fig. 5b, middle column). The actin filaments also showed a stress fiber pattern (Fig. 5b, right column). A similar stress fiber pattern of pMLC, ppMLC and actin was observed in the area of a relatively high cell density, where the lining of VE-cadherin was well developed (Fig. 5c). Collectively, the degree and maturity of cell-cell adhesion appeared to determine which pattern of localization of ppMLC and actin was induced (stress fiber or peripheral bundle).

### Effects of mutations of the phosphorylation sites in MLC on thrombin-induced barrier disruption

**Figure 6:**
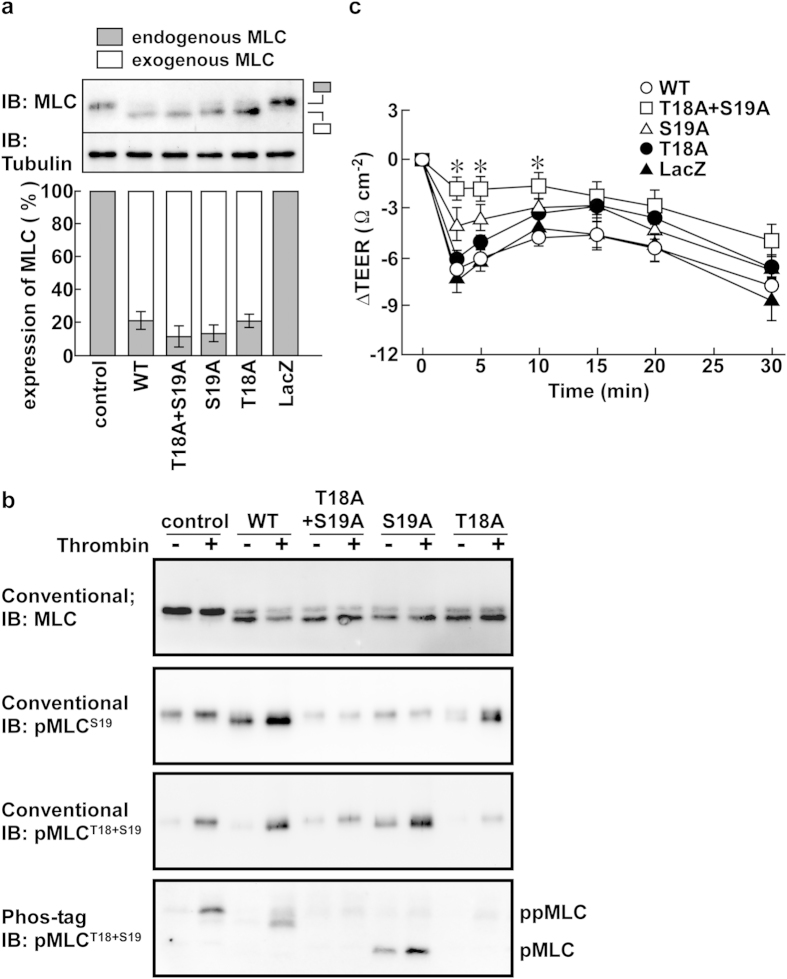
The effects of phosphorylation defective mutants of MLC on the thrombin-induced barrier disruption in PAECs. (**a**) A representative immunoblot (IB) and a summary (n = 4) showing the relative expression levels of endogenous and exogenous MLC in PAECs, expressing wild type (WT) or phosphorylation defective mutants (T18A + S19A, S19A, T18A) of MLC, or LacZ. Control indicates PAECs without adenoviral infection. (**b**) Representative immunoblots (n = 4) obtained for the analysis of the phosphorylation of endogenous and exogenous MLC with the indicated primary antibodies, before (−) and 3 min after (+) stimulation with 1 u ml^−1^ thrombin. (**c**) A summary (n = 7–10) of the temporal changes in the TEER induced by 1 u ml^−1^ thrombin in PAECs expressing exogenous MLC or LacZ, as indicated. The data are the means ± SEM. *P < 0.05 vs. WT, according to an ANOVA followed by Dunnett’s post-hoc test.

In order to obtain further support for the distinct roles for pMLC and ppMLC in the thrombin-induced barrier disruption, the functional effects of mutations of the specific phosphorylation sites in MLC were examined. The human MYL12B isoform of MLC and its mutants were expressed in PAECs using adenoviral expression vectors. The exogenously expressed MLC mobilized faster than the endogenous MLC in conventional SDS-PAGE (Fig. 6a,b). Approximately 80% of the MLC in PAECs was estimated to be derived from the exogenous MLC for all constructs (Fig. 6a). The expression of endogenous MLC was suppressed in the cells expressing exogenous MLC (Fig. 6a,b).

The intended mutations were validated by examining their phosphorylation in response to thrombin stimulation (Fig. 6b). The anti-pan MLC antibody detected doublets in PAECs expressing exogenous MLC (Fig. 6b, uppermost immunoblot). The immunoreactivity of the lower band of wild type MLC to both the anti-pMLC^S19^ and anti-pMLC^T18+S19^ antibodies increased 3 min after thrombin stimulation (Fig. 6b, conventional). The double mutation of T18 and S19 abolished the immunoreactivity of the lower band to the phospho-specific antibodies. For the S19A mutant, the immunoreactivity of the lower band for the anti-pMLC^S19^ antibody was abolished, while immunoreactivity toward the anti-pMLC^T18+A19^ antibody was observed. However, this band exhibited a mobility similar to that for pMLC in Phos-tag SDS-PAGE (Fig. 6b, Phos-tag), indicating that the S19A mutant detected by the anti-pMLC^T18+A19^ antibody was mono-phosphorylated at T18. For the T18A mutant, immunoreactivity of the lower band with the anti-pMLC^S19^ antibody was observed, while the immunoreactivity with the anti-pMLC^T18+S19^ antibody was abolished.

In PAECs expressing wild type MLC, thrombin induced a decrease in the TEER similar to that seen with LacZ-transfected PAECs (Fig. 6c). When both phosphorylation sites (T18 and S19) were mutated to alanine, the thrombin-induced decrease in the TEER was substantially inhibited (Fig. 6c). The T18A mutant had no significant effect on the thrombin-induced decrease in the TEER (Fig. 6c). The S19A mutant exhibited modest inhibitory effects on thrombin-induced barrier disruption, but its effects were not statistically significant (Fig. 6c).

### Localization of MLC mutants in PAECs

**Figure 7:**
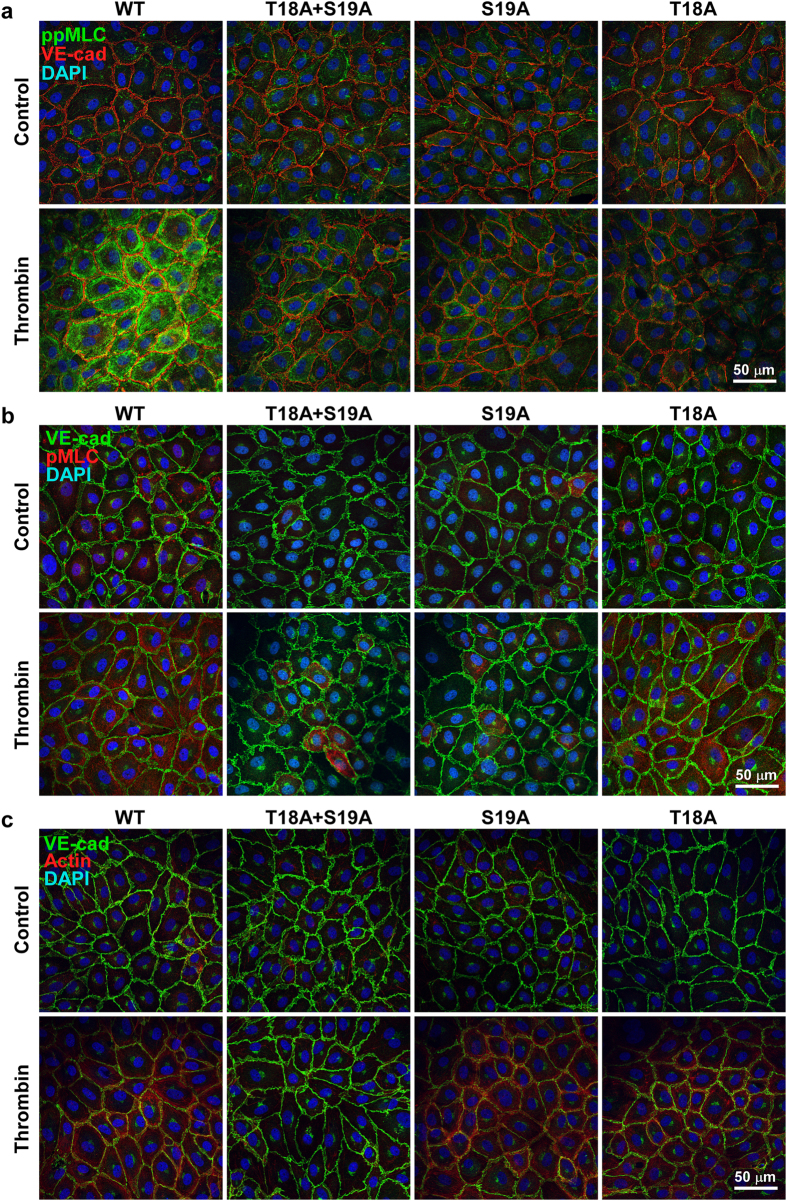
The localization of pMLC, ppMLC and actin 3 min after thrombin stimulation in PAECs expressing exogenous MLC. Representative merged images of confocal microphotographs of the fluorescence staining with the indicated primary antibodies and Texas-red phalloidin in PAECs expressing wild type (WT) or phosphorylation defective mutants (T18A + S19A, S19A, T18A) of MLC, before (Control) and 3 min after stimulation with 1 u ml^−1^ thrombin (Thrombin). pMLC and ppMLC indicate the fluorescence images obtained with the anti-pMLC^S19^ antibody and anti-pMLC^T18+S19^ antibody, respectively. n = 5 (**a**,**b**), n = 2 (**c**).

The subcellular localization of pMLC (i.e., detection with the anti-pMLC^S19^ antibody), ppMLC (i.e., detection with the anti-pMLC^T18+S19^ antibody) and actin in PAECs expressing wild type and mutant MLC was examined before and 3 min after thrombin stimulation (Fig. 7). PAECs expressing wild type MLC exhibited pMLC, ppMLC and actin localizations similar to those seen with control non-infected cells (shown in Fig. 4), both before and 3 min after thrombin stimulation. Namely, ppMLC and actin filaments exhibited a peripheral bundle pattern after thrombin stimulation, while pMLC exhibited a perinuclear cytoplasmic localization (Fig. 7). The T18A+S19A mutant abolished the peripheral localization of ppMLC and actin filaments after thrombin stimulation (Fig. 7a,c). The residual staining with the anti-pMLC^T18+S19^ antibody was conceivably derived from residual endogenous MLC (Fig. 7a). The immunoreactivity with the anti-pMLC^S19^ antibody was also substantially decreased compared to that seen with wild type MLC (Fig. 7b).

In PAECs expressing the S19A or T18A mutants, the immunoreactivity with the anti-pMLC^T18+S19^ antibody was scarcely detected before and after thrombin stimulation (Fig. 7a). Regarding the S19A mutant, this observation was apparently inconsistent with that seen with immunoblots using this antibody, which demonstrated the mono-phosphorylation at T18 in the S19A mutant (Fig. 6b). The reason for this apparent discrepancy remains unclear. The reactivity of the anti-pMLC^T18+S19^ antibody might differ between immunoblotting and immunofluorescence staining. In contrast, the anti-pMLC^S19^ antibody homogeneously bound to the cytoplasm of PAECs expressing the T18A mutant after thrombin stimulation, while this antibody exhibited only residual staining in the cells expressing the S19A mutant (Fig. 7b). These observations seen with the anti-pMLC^S19^ antibody are consistent with those seen in the immunoblot analysis (Fig. 6b). Most important for the functional relevance, the thrombin-induced peripheral actin bundle formation was abolished only by the double mutation. PAECs expressing wild type MLC and the T18A and S19A mutants all exhibited peripheral actin bundles after thrombin stimulation (Fig. 7c). The effects of the MLC mutations on the peripheral actin bundle formation were therefore well correlated with those on the barrier disruption (Fig. 6c).

### Actin stress fiber formation in the late phase after thrombin stimulation

**Figure 8:**
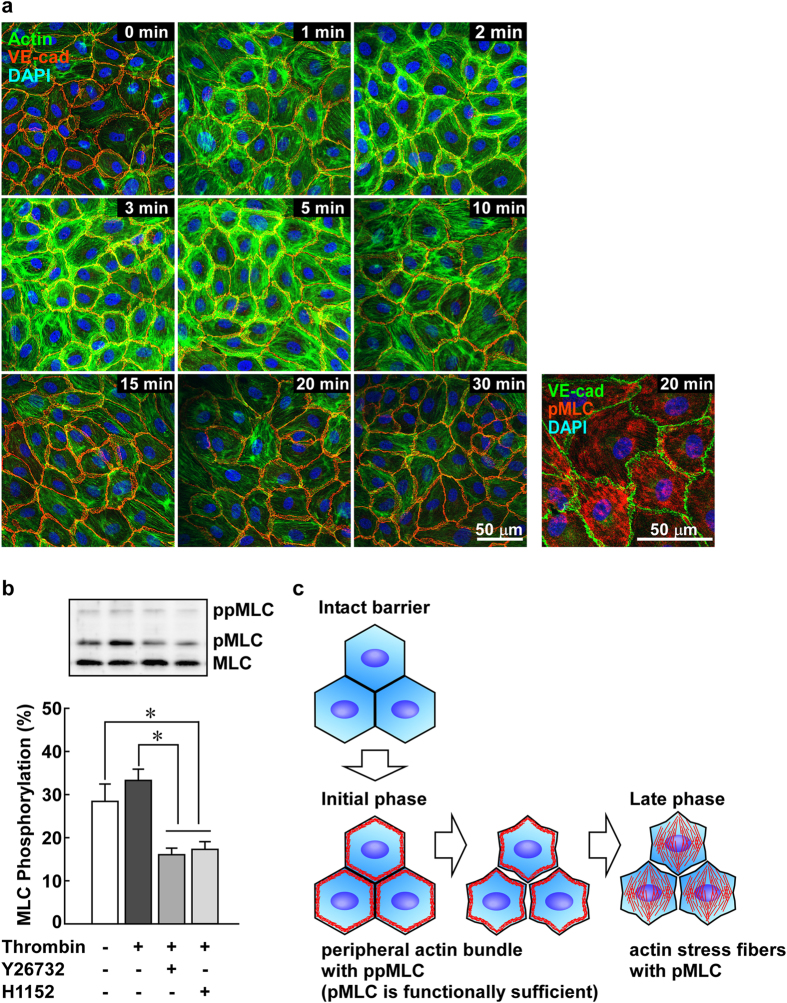
The stress fiber pattern of localization of pMLC and actin in PAECs in the late phase after thrombin stimulation and proposed scheme for the sequential events of barrier disruption. (**a**) Representative merged images of confocal microphotographs of the fluorescence staining of VE-cadherin (VE-cad) and actin (n = 4) or VE-cadherin (VE-cad) and pMLC (n = 3) at the indicated times after stimulation with 1 u ml^−1^ thrombin. (**b**) The effects of Rho-kinase inhibitors, 10 μM Y27632 and 1 μM H1152, on the level of pMLC at 20 min after thrombin stimulation. The inhibitors were applied 10 min after thrombin stimulation, and the levels of pMLC were evaluated 10 min later, i.e., 20 min after thrombin stimulation, with Phos-tag SDS-PAGE. The data are the means ± SEM (n = 5). *P < 0.05, according to an ANOVA followed by the Turkey-Kramer post-hoc test. (**c**) A schematic presentation of the proposed sequential events that occur during endothelial barrier disruption.

Loose or immature cell-cell adhesion predisposes actin filaments to form stress fibers in response to thrombin (Fig. 5). Once peripheral actin bundles are formed in the early phase, the tight cell-cell adhesion might become loosened due to concentric contraction^29^. In this case, actin filaments might be rearranged from peripheral bundles to stress fibers in the later phase. In fact, the time course analysis of actin filament organization revealed that such reorganization was found in the late phase after thrombin stimulation (Fig. 8a). The peripheral actin bundles were observed as early as 1 min after stimulation and up to 5 min after. Thereafter, the actin filaments were rearranged as stress fibers (Fig. 8a). At 20 min after thrombin stimulation, pMLC was localized in a stress-fiber pattern (Fig. 8a). When Rho-kinase inhibitors were applied 10 min after thrombin stimulation, the level of pMLC at 20 min after thrombin stimulation was significantly suppressed Fig. 8b).

## Discussion

The main finding of the present study is that MLC di-phosphorylation and actin bundle formation at the cell periphery play a critical role as initial events during the thrombin-induced barrier disruption. This finding is novel in two respects. First, the present study demonstrated a specific role of ppMLC in barrier disruption, which is distinct from that of pMLC. The temporal profile, subcellular localization and sensitivity to Rho-kinase inhibitors and removal of extracellular Ca^2+^ were different between pMLC and ppMLC. The decrease in the TEER during the early phase of the thrombin-induced barrier disruption (<10 min) exhibited sensitivity to Rho-kinase inhibitors and resistance to extracellular Ca^2+^ removal similar to those seen with ppMLC, but not pMLC. These observations suggest the specific involvement of ppMLC in the early phase of thrombin-induced barrier disruption.

Second, the present study proposes that the peripheral actin bundle formation is an initial event during barrier disruption. Stress fiber formation was observed in the later phase (>10 min). This is a new finding in terms of the previous understanding of the role of stress fiber formation in endothelial barrier disruption^1^,^2^,^5^. Notably, the peripheral actin bundles were temporally and spatially associated with ppMLC. These results suggest that the thrombin-induced barrier disruption starts from ppMLC at the cell periphery, which in turn causes actin bundle formation. This event has been overlooked in the earlier views of the mechanism underlying endothelial barrier disruption.

Distinct functional roles of pMLC and ppMLC have previously been shown for the regulation of myosin ATPase activity^11^,^12^,^21^, filament formation of myosin or actin^18^,^21^,^22^,^23^, cytokinesis^20^, cellular stiffness^17^,^19^, and cell migration^16^, mainly by using either unphosphorylatable (A) or phosphomimetic (D) mutants of MLC at T18 and S19. These differences were sometimes quantitative, but other times were qualitative. However, many reports have shown a quantitative difference in such aspects as the regulation of myosin ATPase activity^11^,^12^,^21^,^23^, cellular stiffness^17^,^19^, filament formation of actin^22^ and stabilization of myosin filaments^16^,^18^. In these cases, ppMLC exerts a similar but greater effect than pMLC. The present study demonstrated the qualitative differences between pMLC and ppMLC. The most striking difference is their subcellular localization. This is in contrast to the findings of many other reports showing the co-localization of pMLC and ppMLC in stress fibers or the contractile ring of cytokinesis^20^,^22^,^30^.

Only a few reports have shown a differential localization of pMLC and ppMLC, and these were in situations unlike those in the present study^16^,^18^. For example, in CHO cells, the adhesion to fibronectin-coated substratum increased the levels of pMLC and ppMLC^16^. Under such situations, pMLC localized in the area of the lamellipodia, while ppMLC localized in the rear or side area. The preferential localization of ppMLC at the region of the cell-cell junction was shown in Madin Darby canine kidney (MDCK) II epithelial cells^18^. However, this peripheral localization of ppMLC appears to be different from that seen in the present study, but it is similar to that of circumferential actin filaments, which are associated with mature cell-cell adhesion^5^. Furthermore, pMLC and ppMLC were shown to localize in different regions of stress fibers in MDCK II cells; pMLC localized in the region where stress fibers were stretching, while ppMLC localized in the region where stress fibers were contracting^18^. The present study thus demonstrate that the thrombin-induced barrier disruption is a new situation where pMLC and ppMLC exhibit differential localization.

In addition to the subcellular localization, pMLC and ppMLC exhibited different kinetics and sensitivities toward kinase inhibitors. These observations suggest that pMLC is not a prerequisite for ppMLC, and that they are differently regulated. It is conceivable that the distinct localization of pMLC and ppMLC is attributable to the distinct localization of the responsible kinases. The Ca^2+^-calmodulin-dependent MLC kinases, MLCK, phosphorylates MLC with a preference for S19 to T18^6^,^10^. Rho-kinase, ZIP kinase and integrin-linked kinases phosphorylate MLC with no preference between T18 and S19^31^,^32^,^33^,^34^,^35^. In the present study, the level of ppMLC was almost completely abolished by Rho-kinase inhibitors, while pMLC was resistant in the early phase and sensitive in the later phase following thrombin stimulation. The Rho-kinase-dependent ppMLC at the cell periphery appears to be consistent with the fact that RhoA is activated at the plasma membranes^15^. Rho-kinase contributes to MLC phosphorylation either by directly phosphorylating MLC^31^,^32^ or by inhibiting MLC phosphatase activity via the phosphorylation of MYPT1, a regulatory subunit of MLC phosphatase, or CPI-17, an inhibitory protein of MLC phosphatase^13^,^14^,^15^. However, precisely how pMLC and ppMLC are regulated during the time course of barrier disruption and the identity of the responsible kinases remain to be determined.

The observations with the mutants of MLC suggest that mono-phosphorylation is functionally sufficient for peripheral actin bundle formation and barrier disruption. The double mutation of T18 and S19 to A was required for the inhibition of both events. This observation gives further support for the correlation between peripheral actin bundle formation and barrier disruption. It also suggests that the localization at the cell periphery, but not the degree of phosphorylation, is essential for the role of ppMLC in barrier disruption. The effects of pMLC and ppMLC on the myosin ATPase activity are quantitatively different^11^,^12^,^21^,^23^. However, the effects of pMLC on the formation of myosin filaments and the sliding velocity of actin filaments are quantitatively similar to those of ppMLC^21^,^23^. These effects of pMLC are consistent with the observations with mutant MLC in the present study.

In the present study, actin stress fiber formation was observed in the late phase (>10 min) during thrombin-induced barrier disruption. However, in the cells examined on early culture days or in the absence of extracellular Ca^2+^, actin stress fiber formation was readily observed as early as 3 min after thrombin stimulation. The degree of maturity of the cell-cell adhesion appears to determine which pattern of actin filaments is induced as an initial event. This is consistent with the notion that the arrangement of actin filaments is subject to the state of VE-cadherin-mediated cell adhesion, the degree of cell density and the nature of the extracellular matrix in cell culture^5^. Subconfluent cultures with an interrupted VE-cadherin lining exhibit many stress fibers^5^. With increasing cell density, the stress fibers disappear and the circumferential actin bundles develop in association with the development of the VE-cadherin lining. Highly confluent endothelial cultures with continuous VE-cadherin lining are characterized by circumferential actin bundles with fewer stress fibers^5^. The notion that the stress fiber formation is a prerequisite for barrier disruption is based on the observations obtained mainly with human umbilical vein endothelial cells or other human endothelial cells^1^,^2^,^5^. Although the inter-endothelial lining of VE-cadherin can be observed at confluence in these cells, it is possible that the maturity of the cell-cell contact remains poor in human endothelial cells. Therefore, stress fiber formation might be preferentially observed during barrier disruption in these cells. In PAECs, the developmental state of the cell-cell contact was heterogeneous on the early culture days. In the areas of a low cell density, the inter-endothelial VE-cadherin lining was poorly developed (Fig. 5b,c). In the areas of a relatively high cell density, the inter-endothelial VE-cadherin lining was well developed. However, thrombin induced stress fiber formation in both cases (Fig. 5b,c). Therefore, the continuous inter-endothelial lining of VE-cadherin does not necessarily indicate the maturity of the cell-cell adhesion. In the cells with the mature cell-cell contacts, thrombin induced the formation of peripheral actin bundles, thereby generating concentric force^29^, which then loosened the inter-endothelial cell-cell adhesion. Once the cell-cell adhesion was loosened, the peripheral actin bundles could be rearranged into stress fibers. This scenario is consistent with the observations of the present study (Fig. 8c).

In conclusion, the present study proposes that ppMLC and peripheral actin bundle formation play specific roles as initial events that occur during the thrombin-induced barrier disruption. The peripheral localization but not the degree of phosphorylation is suggested to be essential for the function of ppMLC as an initial event. This initial event loosens the inter-endothelial junctions, causing not only an increase in paracellular permeability, but also predisposing actin filaments to rearrange into stress fibers during the sustained phase of barrier dysfunction (Fig. 8c). Thus, the uncovered initial event, i.e., peripheral ppMLC and actin bundle formation, could be a missing event that connects the transition of the circumferential actin filaments associated with mature junctions to the actin stress fibers formed during endothelial barrier disruption.

## Methods

### Materials

Thrombin (T7513, bovine plasma) and (−)-blebbistatin were purchased from Sigma (St. Louis, MO, USA). PAR_1_-AP (TFLLR-NH_2_) and PAR_4_-AP (AYPGKF-NH_2_) were purchased from Bachem (Bubendorf, Switzerland). Phos-tag acrylamide (AAL-107) was purchased from Wako pure chemicals (Tokyo, Japan). Y27632 and H1152 Glycyl were purchased from Merck Millipore (Billerica, MA, USA). The primary antibodies used were an anti-pan MLC antibody (sc-15370; Santa Cruz, Dallas, TX, U.S.A.), anti-phospho-MLC at S19 (pMLC^S19^) antibody (#3675; Cell signaling, Beverly, MA, U.S.A.), anti-phospho-MLC at T18 and S19 (pMLC^T18+S19^) antibody (#3674; Cell signaling) and rabbit (#2500, Cell signaling) and mouse monoclonal (sc-9989, Santa Cruz) anti-VE-cadherin antibodies. The secondary antibodies conjugated with horseradish peroxidase were purchased from Sigma. Anti-mouse IgG antibodies conjugated with Alexa488 (#4408) or Alexa647 (#4410) and anti-rabbit IgG antibodies conjugated with Alexa488 (#4412) or Alexa647 (#4414) were purchased from Cell Signaling. Anti-mouse IgG and anti-rabbit IgG antibodies conjugated with Alexa 546 (A11018 and A11071, respectively), FITC-phalloidin and Texas-red phalloidin were purchased from Life Technologies-Molecular Probes Japan (Tokyo, Japan). The PAR_1_ antagonist, E5555, was kindly provided by Eisai Co., Ltd. (Tokyo, Japan)^36^,^37^,^38^.

### Cell culture

Porcine aortic endothelial cells (PAECs) were established from porcine aortas obtained at a local slaughterhouse, as described previously^39^,^40^.The cells were cultured in Dulbecco’s modified Eagle’s medium (Sigma, St. Louis, MO, U.S.A.) supplemented with 10% fetal bovine serum with no antibiotics^41^. Cells at passages 10–16 were used in all investigations. For the experiments, the cells were plated at the density indicated in the following sections, and were cultured for six to seven days until confluent (approximately 7 × 10^4^ cells cm^−2^). The cells were then serum-starved overnight before the day of the experiment. HEK293 cells and HeLa cells were purchased from RIKEN Bio-resource Center (Tsukuba, Japan) and were cultured in Dulbecco’s modified Eagle’s medium supplemented with 10% fetal bovine serum.

### Measurement of the trans-endothelial electrical resistance

The endothelial barrier function was evaluated by assessing the trans-endothelial electrical resistance (TEER). PAECs were plated at 1.5 × 10^4^ cells in 0.32-cm^2^ culture inserts with polyethylene terephthalate membranes (0.4-μm pore size, 1.6 × 10^8^ pores cm^−2^) in 24-well culture plates (BD Falcon, Tokyo, Japan). TEER measurements were conducted in Hepes-buffered saline (HBS: 10 mM Hepes at pH 7.4, 135 mM NaCl, 5 mM KCl, 1 mM CaCl_2_, 1 mM MgCl_2_, 5.5 mM D-glucose) at 37 °C with an EVOM^2^ voltohmmeter equipped with a STX2 electrode (World Precision Instruments, Sarasota, FL USA). The cells were equilibrated in HBS (1 ml in the lower chamber and 0.4 ml in the upper chamber) for 10 min, and then were subjected to the experimental protocol. All reagents were applied to the upper chamber by changing the whole solution. The change in the TEER from the value obtained in HBS just prior to agonist stimulation (ΔTEER) was expressed as an absolute value of the resistance normalized by the surface area of the culture insert membrane (Ω cm^−2^), unless otherwise specified. There were no significant changes in the TEER during the 10-min equilibration period in HBS.

### Preparation of cell lysates for the analysis of myosin light chain phosphorylation

The cells were plated at 3 × 10^5^ cells in 60-mm culture dishes. On the experimental day, the cells were rinsed three times with pre-warmed phosphate-buffered saline (PBS; 136.9 mM NaCl, 2.7 mM KCl, 8.1 mM Na_2_HPO_4_, 1.47 mM KH_2_PO_4_), and then equilibrated for 10 min and subjected to the experimental protocols in HBS at 37 °C. At each time point, the cells were rinsed once with ice-cold PBS containing 0.1 mM EDTA to remove residual extracellular Ca^2+^. After the wash buffer was thoroughly removed, the cells on dishes were lysed in 70 μL ice-cold lysis buffer containing 50 mM Hepes, 150 mM NaCl, 0.5% Nonidet P-40, 1 mM dithiothreitol, 10 μg ml^−1^ leupeptin, 10 μg ml^−1^ aprotinin, 10 μM 4-amidinophenylmethanesulfonyl fluoride (p-APMSF), 20 μM NaF, 1 mM Na_3_VO_4_, 50 μM calpain inhibitor I and 5 μM microcystin-LR. The lysates were immediately collected using a cell scraper, transferred to a microcentrifuge tube and snap-frozen in liquid N_2_. The lysates were kept at −80 °C until use. The lysates were then thawed on ice for 20 min, and centrifuged at 12,000 rpm for 20 min on microcentrifuge (Type 1120; Kubota, Tokyo, Japan) in a cold room. The protein concentrations of supernatants were estimated with a Coomassie (Bradford) protein assay kit (Thermo Scientific Pierce, Rockford, IL, USA). The lysates were prepared for SDS-PAGE by adding an appropriate volume of 6x Laemmli’s sample buffer and β-mercaptoethanol to a final concentration of 5%.

### Phos-tag and conventional SDS-PAGE, and the immunoblot analysis of MLC phosphorylation

The samples (3.5 μg proteins) were subjected to SDS-PAGE on 10% polyacrylamide gels containing 30 μM Phos-tag and 60 μM MnCl_2_ in Phos-tag SDS-PAGE^26^,^42^. After electrophoresis, the gels were soaked for 30 min at room temperature in transfer buffer (25 mM Tris-hydroxymethyl aminomethane, 192 mM glycine, 20% methanol) containing 2 mM EDTA to remove Mn^2+^, and then in the transfer buffer for 15 min. In conventional SDS-PAGE, the proteins were separated on 12.5% polyacrylamide gels, with no Phos-tag or MnCl_2_, in triplicate for the detection of total MLC, pMLC and ppMLC. The Mn^2+^ removal step was omitted in conventional SDS-PAGE. Proteins were then transferred to polyvinylidene difluoride membranes (Merck Millipore). The membranes were post-fixed in PBS containing 0.5% formaldehyde for 45 min^42^, then rinsed once with PBS containing 0.1% Tween-20 (T-PBS), and blocked with 5% non-fat dry milk in T-PBS overnight in a cold room. The membranes were incubated with primary and then secondary antibodies diluted in Can-Get-Signal immunoreaction enhancer solution (Toyobo, Osaka, Japan) for 60–90 min at room temperature. The primary antibodies and their dilutions were: anti-pan MLC antibody (×2,000), anti-pMLC^S19^ antibody (×2,000 – ×5,000) and anti-pMLC^T18+S19^ antibody (×2,000). The immune complexes were detected with an ECL Plus or ECL Select detection kit (GE Healthcare, Buckinghamshire, UK).

The chemiluminescence emission was captured with a ChemiDoc XRS-J instrument and was analyzed using the Quantity One software program (Bio Rad, Hercules, CA, USA). The image capture time was adjusted to keep the intensity of image below the maximal saturation level of the system. In the Phos-tag SDS-PAGE analysis, the percentages of pMLC and ppMLC in the total MLC (a sum of the optical densities of non-phosphorylated MLC, pMLC and ppMLC), all of which were detected with the anti-pan MLC antibody, were calculated for each lane. In the conventional SDS-PAGE analysis, the density of the pMLC and ppMLC bands was normalized to the density of the corresponding pan-MLC band, then the levels of pMLC and ppMLC were expressed as a fold-increase from the prestimulation level.

### Immunofluorescence staining

The cells were plated at 1.5 × 10^5^ cells on glass coverslips (22 × 22 mm, Matsunami, Osaka, Japan) in 35-mm culture dishes. For all experiments, the cells were rinsed three times with pre-warmed PBS, and then equilibrated for 10 min and subjected to the experimental protocols in HBS at 37 °C. At each time point, the cells were fixed with 4% formaldehyde in PBS for 20 min, and permeabilized by a 5-min incubation in 0.05% Triton X100-containing PBS. After 60-min of blocking with 5% bovine serum albumin in PBS, the cells were incubated with the indicated combinations of primary antibodies diluted in Can Get Signal Immunostain (Toyobo) for 60 min at room temperature and then with the appropriate combinations of fluorescent dye-conjugated secondary antibodies for 60 min at room temperature. For staining of the actin filaments, the specimens were incubated with either 5 units ml^−1^ FITC- or TEXAS-red phalloidin in PBS for 20 min after the immunostaining procedures. The cells were then mounted in ProLong Gold antifade reagent with DAPI (Life Technologies-Molecular Probe). The confocal fluorescence images were captured with a Nikon confocal laser microscope A1 with a 60x objective lens (Tokyo, Japan). The original images were saved in the JPEG2000 format using an imaging software program, NIS-elements AR (Nikon). The images used for presentation were exported from the original files and saved in the TIFF format using a Snapshot function of the software. The intensity of the red, green or blue channels was set to 0 using the Photoshop software (Adobe, San Jose, CA, USA) to show the remaining two colors in TIFF files.

### Construction of adenoviral vectors

The reverse transcription-polymerase chain reaction (RT-PCR) analysis detected the expression of MYL12B, but not MYL12A or MYL9, as phosphorylatable MLC in PAECs. In brief, total RNA was prepared by using an RNeasy Mini Kit (Qiagen, Hilden, Germany), and the RT reaction was performed with a ReverTra Plus kit and random primers (Toyobo). The PCR reaction was performed with a DNA polymerase, KOD plus ver. 2 (Toyobo). The following primers based on the sequences of human MLC were used for the PCR reaction: AgC CAA g
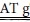
TC CAg CAA gC (forward primer for MYL9), TCA ggg ggC Tgg ggT ggC CT (reverse primer for MYL9), CAC C
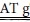
TC gAg CAA AAg AA (forward primer for MYL12A), Tgg AAT TTg AAg TTA TTT CA (reverse primer for MYL12A), CAC C
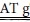
TC gAg CAA AAA gg (forward primer for MYL12B) and TTT TAg CTA AAg TTC TTT CA (reverse primer for MYL12B). The double underlined ATG sequences indicate initiation codons. The reverse primers were designed to include a stop codon or a 3′ region from the stop codon. Therefore, the PCR products covered the full coding region of each isoform. HeLa cells were used as a positive control for the RT-PCR analysis. The expression of all three isoforms were detected in HeLa cells, and the sequences of PCR products were confirmed. Since this preliminary study determined that MYL12B was a major isoform of phosphorylatable MLC expressed in PAECs, this isoform was used in the subsequent investigations.

The full length cDNA for human MLY12B was obtained from the total RNA of HeLa cells by RT-PCR as described above. The cDNAs for MYL12B with mutations of T18A and S19A were obtained by PCR cloning using the following long forward primers, which contained the indicated mutations, in combination with the aforementioned reverse primer for MYL12B: C ACC 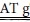 TCg AgC AAA AAg gCA AAg ACC AAg ACC ACC AAg AAg CgC CCT CAg CgT gCA gCA TCC AAT gTg TTT gCC (T18A), C ACC 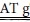 TCg AgC AAA AAg gCA AAg ACC AAg ACC ACC AAg AAg CgC CCT CAg CgT gCA ACA gCC AAT gTg TTT gCC (S19A) and C ACC 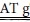 TCg AgC AAA AAg gCA AAg ACC AAg ACC ACC AAg AAg CgC CCT CAg CgT gCA gCA gCC AAT gTg TTT gCC (T18A + S19A). The double underlined ATG indicates an initiation codon. The single underlined sequences indicate mutated codons. Please note that the initiation methionine was removed from the mature MLC^6^,^8^.

Adenoviral expression vectors and virus particles were prepared using an Adenovirus Expression Vector Kit (Takara, Otsu, Japan). The RT-PCR products of MYL12B and its mutants were subcloned into a cosmid vector, pAxCAwtit. The control LacZ construct was provided in the kit. Adenovirus particles were prepared according to the manufacturer’s instructions. The forth preparation of adenovirus particles was used to infect the PAECs. The titers of the preparations were as follows: 3.1 × 10^10^ pfu ml^−1^ (wild type), 2.1 × 10^10^ pfu ml^−1^ (T18A + S19A), 2.4 × 10^10^ pfu ml^−1^ (S19A), 9.0 × 10^9^ pfu ml^−1^ (T18A), and 2.7 × 10^10^ pfu ml^−1^ (LacZ).

### Adenoviral infection and experimental protocol

PAECs were plated as described above. On day 4, the cells were exposed to viral particles for 1 hr at the following MOI: 2.5 × 10^3^ (wild type), 1.7 × 10^3^ (T18A + S19A), 1.9 × 10^3^ (S19A), 7 × 10^2^ (T18A) and 2.2 × 10^3^ (LacZ). Three days after infection, the cells were subjected to an evaluation of the TEER, MLC phosphorylation and immunofluorescence staining. The staining with 5-bromo-4-chloro-3-indolyl-β-D-galactoside (X-gal) of LacZ-infected PAECs indicated that β-galactosidase was expressed in 100% of the population^43^.

### Statistical analysis

The data are the means ± SEM of the indicated number of independent experiments. The StatView ver.5 software program (SAS Institute, Cary, NC, USA) was used to evaluate the statistical significance by analysis of variance (ANOVA), followed by Dunnett’s post-hoc test for comparisons to the controls and Turkey-Kramer test for multiple comparisons. A value of *P* < 0.05 was considered to be statistically significant.

## Additional Information

**How to cite this article**: Hirano, M. and Hirano, K. Myosin di-phosphorylation and peripheral actin bundle formation as initial events during endothelial barrier disruption. *Sci. Rep.* **6**, 20989; doi: 10.1038/srep20989 (2016).

## Supplementary Material

Supplementary Information
