# Supplementary material for: Myosin di-phosphorylation and peripheral actin bundle formation as initial events during endothelial barrier disruption
Source: Sci Rep. 2016 Feb 11;6:20989. doi: 10.1038/srep20989 (PMC4750094; doi:10.1038/srep20989)

Supplementay information

Myosin di-phosphorylation and peripheral actin bundle formation as initial events during endothelial barrier disruption

Mayumi Hirano[1] and Katsuya Hirano[2,*]

[1] Department of Molecular Cardiology, Research Institute of Angiocardiology, Graduate School of Medical Sciences, Kyushu University

[2] Department of Cardiovascular Physiology, Faculty of Medicine, Kagawa University

Legend for Supplementary Figure S1

The effects of blebbistatin on the thrombin-induced changes in the TEER and MLC phosphorylation in PAECs.

(**a**) The effects of 50 μM (n = 4) and 100 μM (n = 7) blebbistatin on the temporal changes in the TEER induced by 1 u ml^{-1} thrombin (Control; n = 7). (**b**) A representative immunoblot (IB) and a summary (n = 4) of the levels of pMLC and ppMLC obtained before (-) and 3 min after (+) stimulation with 1 u ml^{-1} thrombin, in the absence and presence of 100 μM blebbistatin. PAECs were exposed to blebbistatin 30 min prior to and during thrombin stimulation. The data are the means ± SEM. ns, not significantly different; *, $P < 0.05$ (vs. Control in **a**), according to an ANOVA followed by Dunnett’s post-hoc test (**a**) and the Turkey-Kramer test (**b**).

Supplementary Figure S1

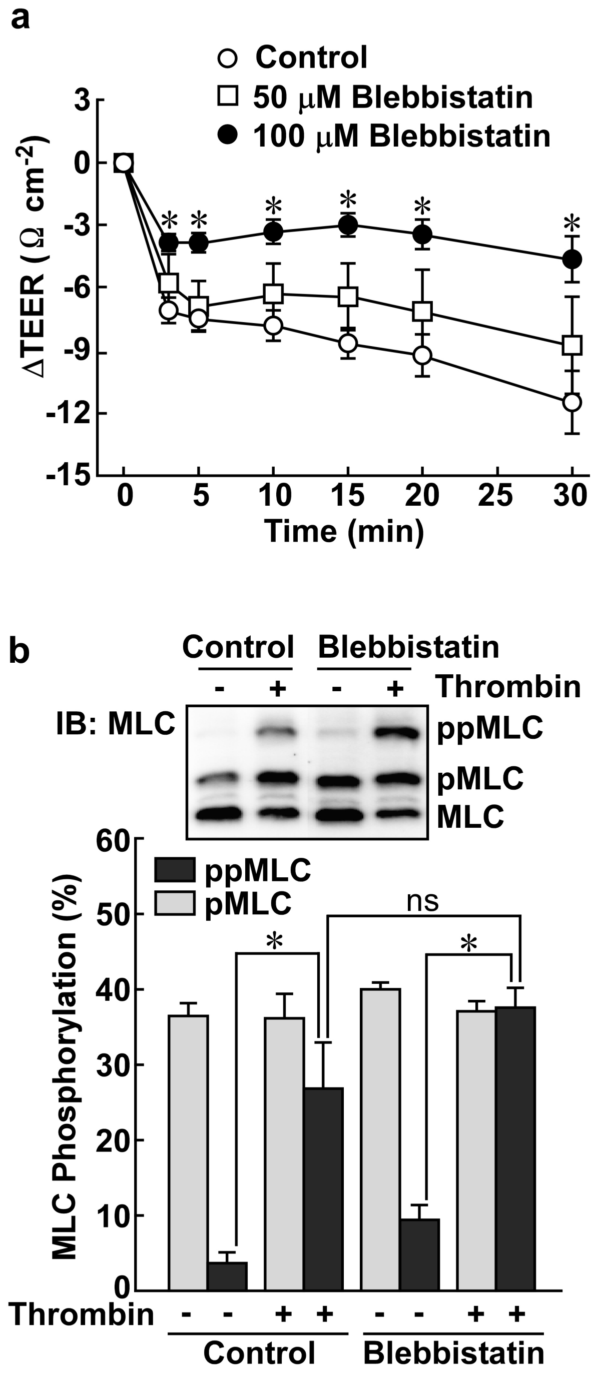

Supplement: Supplementary Information [file srep20989-s1.pdf]
